# Transplantation of Tissue from Native and Cryopreserved Pekin Duck Ovaries to Mulard Ducks with Study of Hormonal Changes After Grafting

**DOI:** 10.3390/ani15162401

**Published:** 2025-08-15

**Authors:** Kitti Buda, Barbara Vegi, Eva Kissne Varadi, Arpad Drobnyak, Eva Török, Zsuzsa Szabo, Bianka Babarczi, Istvan Lehoczky, Krisztina Liptoi

**Affiliations:** 1 National Centre for Biodiversity and Gene Conservation, Institute for Farm Animal Conservation, H-2100 Godollo, Hungary; vegi.barbara@nbgk.hu (B.V.); varadi.eva@nbgk.hu (E.K.V.); drobnyak.arpad@nbgk.hu (A.D.); eva.torok@nbgk.hu (E.T.); szabo.zsuzsa@nbgk.hu (Z.S.); lehoczky.istvan@nbgk.hu (I.L.); liptoi.krisztina@nbgk.hu (K.L.); 2 Institute of Genetics and Biotechnology, Hungarian University of Agriculture and Life Sciences, Szent István Campus, H-2100 Godollo, Hungary; bianka.babarczi@uni-mate.hu

**Keywords:** gonadal tissue transplantation, Mulard duck, gene conservation

## Abstract

Orthotopic transplantation of ovarian tissue at one day of age is a promising solution for preserving female genetic material in avian species; using sterile recipients can ensure that all offspring are donor-derived. This study focuses on the suitability of the Mulard duck as a sterile recipient for Pekin duck donors and provides an investigation of the hormonal background. Firstly, native Pekin ovarian tissue was grafted into Mulard duck recipients, resulting in a 40% adhesion rate and follicular development in 50% of the adhered grafts. Secondly, the transplantation of cryopreserved ovarian tissue resulted in a 66% adhesion rate, with 33% of the adhered grafts showing follicular development. Ovulation occurred in 16% of the recipients with adhered grafts, but the eggs did not move into the oviduct. Estrogen levels were elevated in the recipients with adherence but were lower than in the control Pekin group, while progesterone levels remained unchanged. Consequently, recipients received buserelin acetate, a GnRH analogue, to stimulate follicular and oviductal activity. In this group, graft adhesion occurred in 31% of animals, and primordial follicle development in 25%. The hormonal levels of the recipients with adhered ovaries were elevated, but the GnRH analogue treatment did not affect the ovulation process. We conclude that while the Mulard duck shows potential as a sterile recipient in ovarian transplantation, several questions remain unanswered regarding the adequacy of follicular maturation and ovulation.

## Simple Summary

Specialized methods are required to preserve female genetic material in bird gene conservation. By transplanting the ovarian tissue of an indigenous breed into a recipient, 100% of the donor genotype can be regained. Sterile recipients can be used to ensure that offspring is derived from the donor ovary. This study focuses on whether the Mulard duck, a sterile hybrid, can act as a recipient for ovarian tissue from Pekin ducks. Three experiments were conducted. In the first, fresh ovarian tissue was transplanted into Mulard ducks. About 40% of the tissue attached, 50% of which showed early signs of egg development. In the second experiment, frozen (cryopreserved) tissue was used; 66% of the tissue attached, 33% of which showed development. Some ducks ovulated, but the eggs did not move into the oviduct. The third experiment tested a hormone treatment to boost egg development. In this case, 31% of the tissue attached, 25% of which showed early development. Hormone levels were elevated when tissue was attached, but the treatment did not make a difference. We conclude that Mulard ducks could be useful for preserving duck breeds through ovarian transplantation. However, more research is needed to better control the hormones involved in egg-laying.

## 1. Introduction

Current research into in vitro poultry gene conservation is focusing on methods other than semen cryopreservation. In avian species, the male is the homogametic gender and the female is heterogametic, but due to the inability to freeze eggs and embryos, alternative methods for preserving the female genome are required. One promising, well-established modern approach is the cryopreservation and orthotopic transplantation of gonadal tissue in freshly hatched chicks, allowing donor-derived offspring to be regained in the F1 generation [1,2,3].

However, this method is limited by the fact that the recipients’ gonads cannot be fully removed without causing lethal injury due to their anatomical location (near the aorta and vena cava); therefore, both donor- and recipient-derived progeny may result. The use of an infertile recipient can ensure that all offspring are donor-derived. There are sterile hybrids in which only males occur, such as domestic chicken–guinea fowl hybrids [4], while in the cases of pheasant–domestic chicken hybrids [5] and the Mulard duck [6], the male and female sexes can be separated by phenotype. The Mulard duck is the sterile hybrid of the male Muscovy duck (*Cairina moschata*) and the female Pekin duck (*Anas platyrhynchos domesticus*). Females and males can be easily distinguished at day-old age using eye colour and head feathers. The necropsy of a 17-week-old female Mulard duck demonstrated that in spite of their sterility, female Mulard ducks possess an anatomically normal but undeveloped non-functional ovary and oviduct [7].

Ovulation and oviposition are complex, neuroendocrine-regulated mechanisms controlled by the ovary and the brain. The hypothalamus secretes GnRH (gonadotropin-releasing hormone), which induces the hypophysis to release FSH (follicle-stimulating hormone) and LH (luteinizing hormone). FSH and LH stimulate ovarian progesterone and estrogen production, providing feedback to the hypothalamus. Exogenous GnRH analogues affect the release of hypophyseal gonadotropins by interfering with GnRH receptors. GnRH analogues are commonly used for the induction and synchronization of ovulation, the treatment of reproductive disorders, and increasing reproductive activity in mammals [8,9]. In avian species, TETRA-SL non-laying hens were injected with varying doses of buserelin acetate to induce egg production. The GnRH analogue treatment increased egg production, progesterone, estrogen, LH and FSH levels, and follicular development [10]. Elsewhere, the oral administration of a GnRH analogue (LHRH-A2) in Pekin ducks resulted in increased egg production and progesterone and estradiol levels [11].

The aim of our investigations was to determine whether the Mulard duck is a suitable recipient for grafted donated Pekin duck ovarian tissue, as well as to explore the hormonal background.

## 2. Materials and Methods

### 2.1. Institutional Review Board Statement

The animal study protocol was approved by the Institutional Ethics Committee of the National Centre for Biodiversity and Gene Conservation Institute for Farm Animal Conservation (approval number: 5/2021, approval date 5 July 2021) for studies involving animals.

The applied methods were approved by the Directorate of Food Safety and Animal Health of the Government Office of Pest County, Hungary (PE/EA/674-7/2021).

Institutional Review Board Statement: Authorization number of the animal experimentation facility (National Centre for Biodiversity and Gene Conservation Institute for Farm Animal Conservation): 13/2015.

### 2.2. Animals

In this study, Hungarian Pekin ducks from the National Centre for Biodiversity and Gene Conservation (NBGK) gene bank stock and Mulard ducks from Orvia Hungary Ltd. (Sukosd, Hungary). were used. The white variant of Hungarian ducks is more commonly occurring compared to the spotted and brown variants, but in both variants the beak and legs are orange. Adult drakes weigh from 2.50 kg to 3.20 kg, while adult layers weigh from 2.30 kg to 3.00 kg. Yearly egg production is 200.

The Mulard duck is an interspecific hybrid of the male Muscovy duck (*Cairina moschata*) and female Pekin duck (*Anas platyrhynchos domesticus*). The feather colour is white in both sexes, the beak is pale pink, and the legs are orange. The average weight of adult Mulard ducks ranges from 3.6 kg to 4.5 kg.

Orthotopic transplantation of ovarian tissue was performed between Pekin duck donors and Mulard duck recipients. Both the donor and recipient animals were less than 24 h old, and before the surgery, water was provided ad libitum. The average body weight of ranged from 42 g to 55 g in the day-old Pekin ducks and from 42 g to 58 g in the Mulard ducks.

### 2.3. Experiment Design

**Figure 1:**
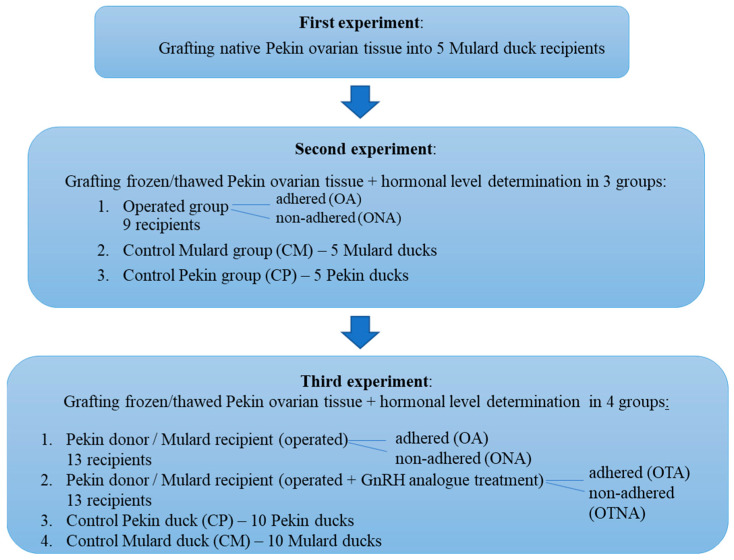
Experimental design of ovarian tissue transplantation in Pekin duck donor/Mulard duck recipient combination.

Firstly, native Pekin duck ovarian tissue was grafted into the Mulard duck recipients. Based on the results of the first experiment, in the second experiment, in addition to grafting cryopreserved ovarian tissue in the same donor/recipient combination, progesterone and estradiol levels were determined in the recipients and in the control Mulard and Pekin ducks. According to the outcome of the second experiment, in the third experiment, after the previously described grafting of cryopreserved tissue and determination of hormonal levels, GnRH analogue treatment was also applied. The design of the experiment is presented in Figure 1.

### 2.4. Orthotopic Transplantation of Native and Cryopreserved Ovarian Tissue, and Postoperative Care

The preparation of the donor gonads, cryopreservation, and thawing were performed according to a technique previously developed in geese [3]. The technique was slightly modified in this study because sterile recipients were used; therefore, in contrast to the original technique, an ovariectomy was not performed in the recipient Mulard ducks. The recipients were anaesthetized according to the above method (a combination of ketamine (15 mg/kg), xylazine (3 mg/kg) and midazolam (2 mg/kg) administered partly intramuscularly and partly intravenously, and, if necessary, an additional Isoflurane mask); next, the gastrointestinal tract was pushed aside, and one piece of native or frozen thawed ovarian tissue (measuring approximately 1 × 2 mm) was grafted next to the recipient’s gonads, near the adrenal gland, aorta, and vena cava. After grafting, the abdominal organs were put back in their anatomical places and the incision was closed in 2 layers using polyglycolic absorbable surgical suture (Safil C 4/0, Braun Ltd. Budapest, Hungary).

After the surgical procedure, 0.06 mg dexamethasone per recipient was administered intramuscularly to prevent acute immune reaction. Mycophenolate mofetil (4 mg/kg) was given per os for two weeks individually as a long-term immunosuppressant. The recipients were housed in groups on straw litter, with food and water provided ad libitum and the temperature and light programmed according to breeding technology standards. A necropsy was performed on the recipients was performed at 52 weeks of age, and the Mulard duck ovary and the donor ovary were identified with a morphological examination.

### 2.5. Hormonal Level Determination

To determine the progesterone and estradiol levels of the animals in both groups, 2 mL of blood samples were collected every second week from the vena brachialis of each animal between 18 and 52 weeks of age into heparine tubes (VACUETTE Lithium-heparine tubes, Greiner Bio-One GMBH, Kremsmünster, Austria). After centrifugation at 4 °C and 3000 rpm for 20 min, 1 mL serum was stored in Eppendorf tubes at −70 °C. Progesterone and estradiol levels were determined using NOVATEC Progesterone and 17-beta-estradiol ELISA kits (Novatec Immundiagnostica GMBH, Dietzenbach, Hungary).

### 2.6. GnRH Analogue Treatment

Buserelin acetate (Receptal, MSD Animal Health, Intervet Hungary Ltd. Budapest, Hungary) treatment was performed in the Pekin duck donor/Mulard duck recipient group twice over a 4-day period at 22 weeks of age through 0.2 mL/recipient intramuscular injection.

### 2.7. Statistical Analysis

Since the data did not follow a normal distribution due to the large standard deviations, we selected our statistical methods accordingly. (Tibco Statistica, version 13.5.0.17). Group comparisons were performed using the Kruskal–Wallis ANOVA and Median test. If a difference was found based on these, we conducted further analysis using the Kolmogorov–Smirnov two-sample test. Changes within groups were analyzed using the Friedman ANOVA and Kendall’s concordance method. If differences were detected, we applied the Wilcoxon matched-pairs test for further analysis.

## 3. Results

### 3.1. First Experiment—Transplantation of Native Ovarian Tissue Between Pekin Duck Donors/Mulard Duck Recipients

**Figure 2:**
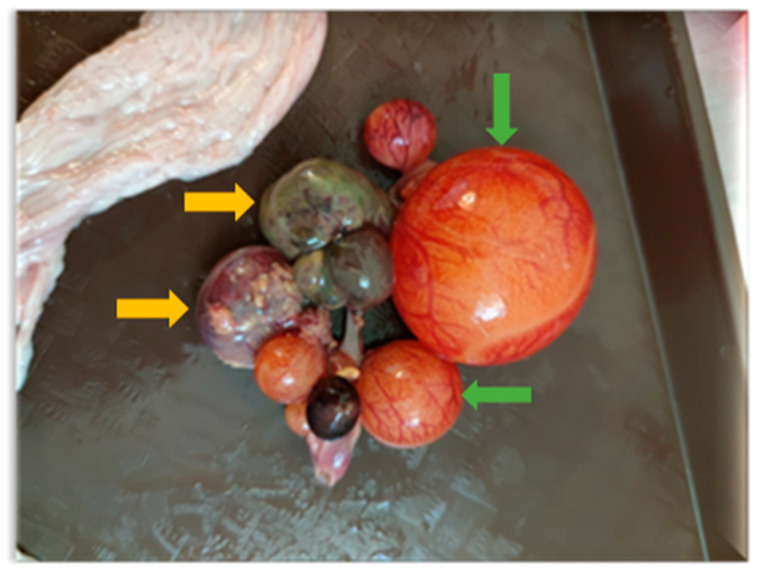
Follicle development beginning in adhered donor ovary. Developing follicles are marked with green arrows and atrophied follicles with yellow arrows.

Necropsies of the recipient Mulard ducks were performed at 52 weeks of age. It was found that 40% of the grafted ovarian tissue adhered close to the recipients’ ovaries. The recipients’ ovaries and oviducts seemed undeveloped and non-functional and follicular development had begun in 50% of the adhered donor ovarian tissue (Figure 2).

### 3.2. Second Experiment—Transplantation of Cryopreserved Ovarian Tissue Between Pekin Duck Donors/Mulard Duck Recipients with Determination of Hormonal Levels

#### 3.2.1. Results of the Necropsy

**Figure 3:**
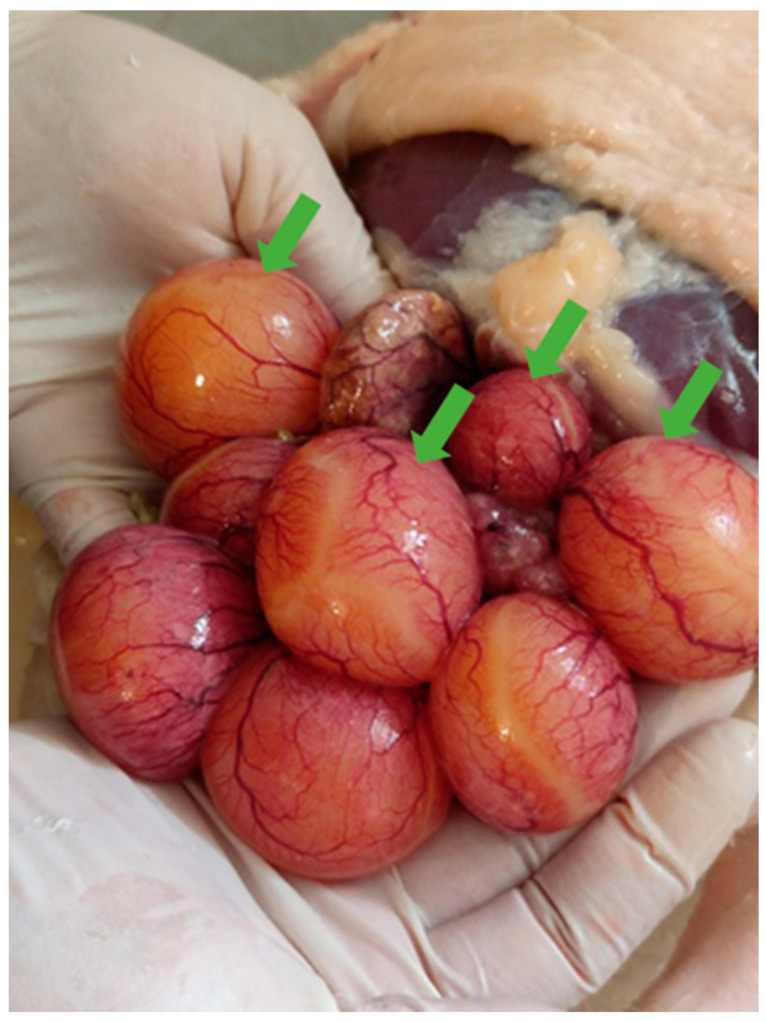
Follicular development (green arrows) on adhered donor ovary in Mulard duck recipient at 52-week autopsy.

**Figure 4:**
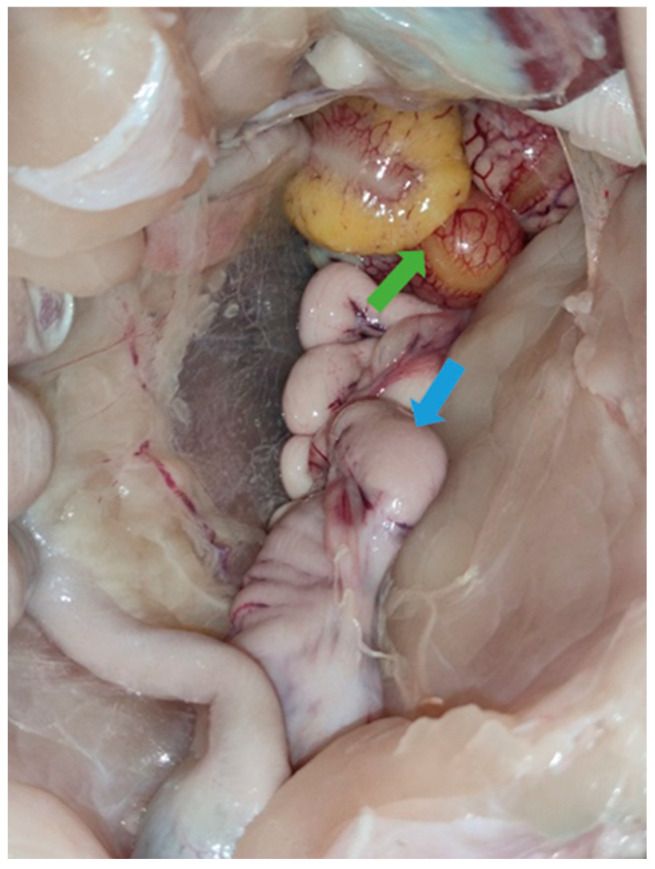
Follicular development (green arrow) on adhered donor ovary and developed oviduct (blue arrow) of 52-week-old Mulard recipient.

**Figure 5:**
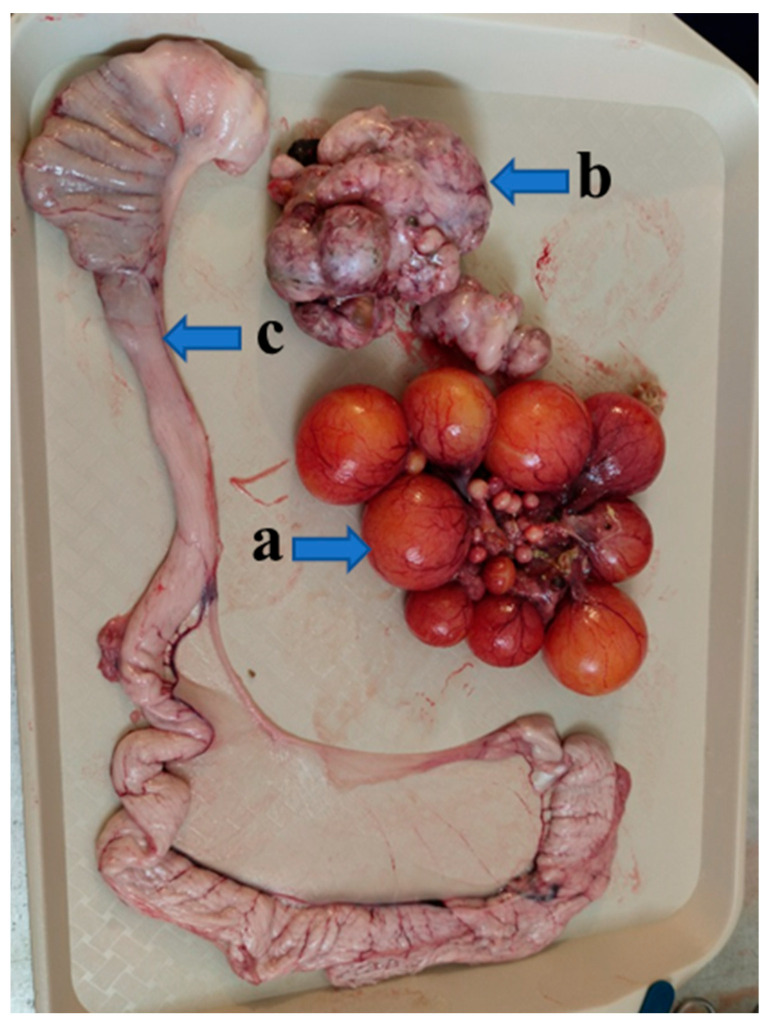
The adhered donor ovary (a) of a Mulard duck recipient with follicles at different developmental stages, the recipient’s own atrophied ovary (b), and the developed oviduct (c) at 52 weeks of age.

**Figure 6:**
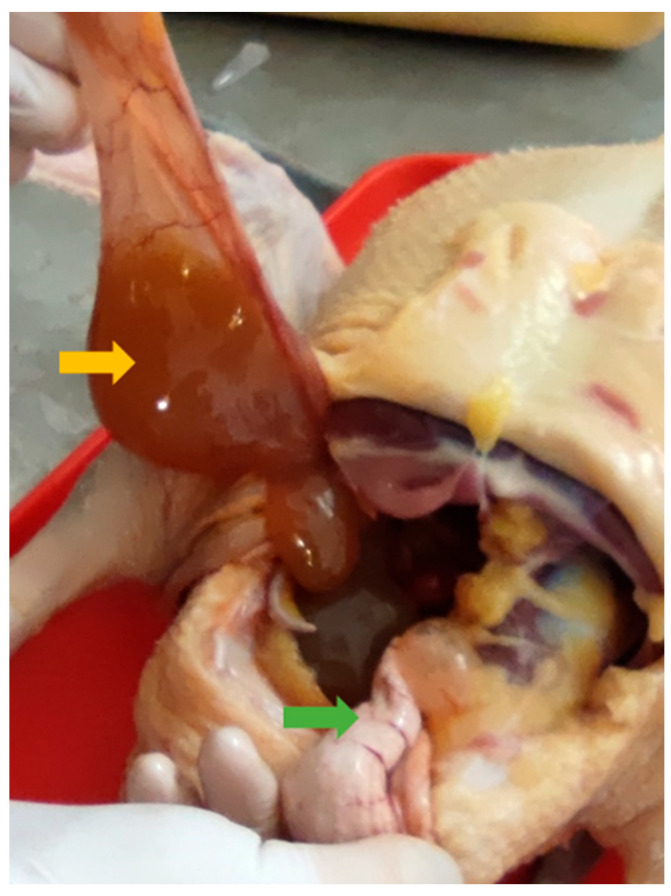
Ovulated egg moved into a different serous membrane capsule (yellow arrow) instead of the oviduct (green arrow).

Necropsies of the recipient Mulard ducks were performed at 52 weeks of age, with the adhesion rate of the grafted Pekin duck ovarian tissue found to be 66%. In the animals with adhered donor gonads, follicular development was found in 33% (Figure 3 and Figure 4). The recipients’ ovaries were atrophied (Figure 5). The oviducts of the animals with mature follicles were also developed; the physical appearance was similar to that of a layer in production (Figure 5 and Figure 6). Ovulation occurred in 16% of the recipients with adhered donor ovaries, but the eggs moved into a serous membrane capsule and not the oviduct (Figure 6).

#### 3.2.2. Results of the Hormonal Level Determination

**Figure 7:**
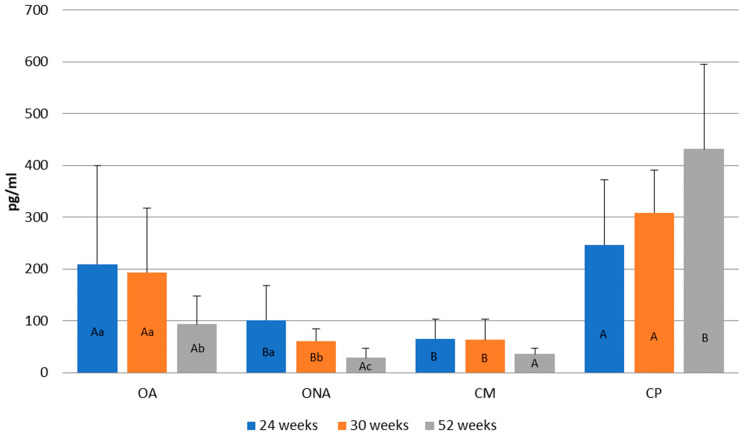
Comparison of estrogen levels at 24, 30, and 52 weeks of age in operated Mulard recipients with adhered donor ovary (OA) and non-adhered donor ovary (ONA), and control Mulard (CM) and control Pekin (CP) groups. A, B: *p* ≤ 0.05: significance between the groups. a, b, c: *p* ≤ 0.05: significance within the groups.

Figure 7 shows the changes in estrogen levels at different weeks of age. Compared to the control Pekin group (CP) and the operated adhered group (OA), lower estrogen levels were observed in the operated non-adhered (ONA) and control Mulard (CM) groups. At weeks 24 and 30, the estrogen levels in the operated adhered group (OA) were not significantly lower compared to those in the Pekin group.

In the operated adhered group (OA), estrogen levels significantly decreased by week 52. In the operated non-adhered group (ONA), a decrease was also observed by week 52. In the control Mulard group (CM), no significant difference was found between the three measurements. In the control Pekin group (CP), although estrogen levels increased, there was no significant difference by week 52.

**Figure 8:**
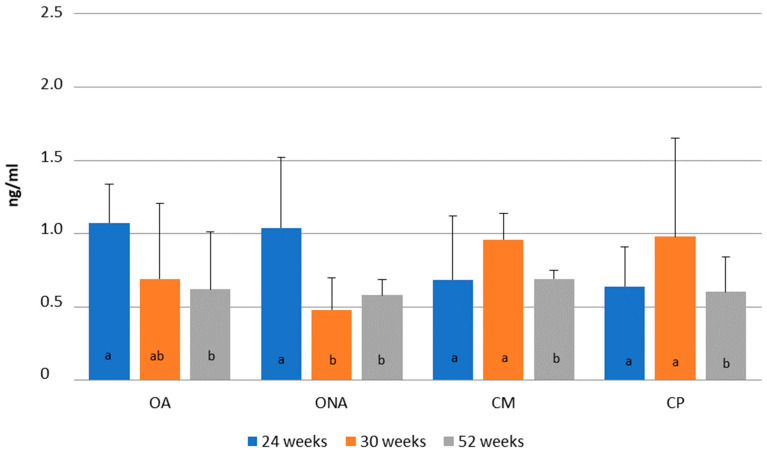
Comparison of progesterone levels at 24, 30 and 52 weeks of age in operated Mulard recipients with adhered donor ovary (OA) and non-adhered donor ovary (ONA), and in the control Mulard (CM) and control Pekin (CP) groups. a, b: *p* ≤ 0.05: significance within the groups.

In Figure 8, changes in progesterone level are presented according to weeks of age. In the two operated groups (OA, ONA), the highest progesterone level was observed at 24 weeks of age (OA: 1.07 ng/mL; ONA: 1.03 ng/ML), while in the control groups (CM, CP), the highest level was observed at 30 weeks of age (CM: 0.95 ng/mL; CP: 0.97 ng/mL). A significant decrease in progesterone levels occurred in the operated non-adhered group (ONA). No differences were found in progesterone levels between the groups.

### 3.3. Third Experiment—Transplantation of Cryopreserved Ovarian Tissue Between Pekin Duck Donors/Mulard Duck Recipients, GnRH Analogue Treatment, and Hormonal Level Determination

#### 3.3.1. Results of the Necropsy

**Figure 9:**
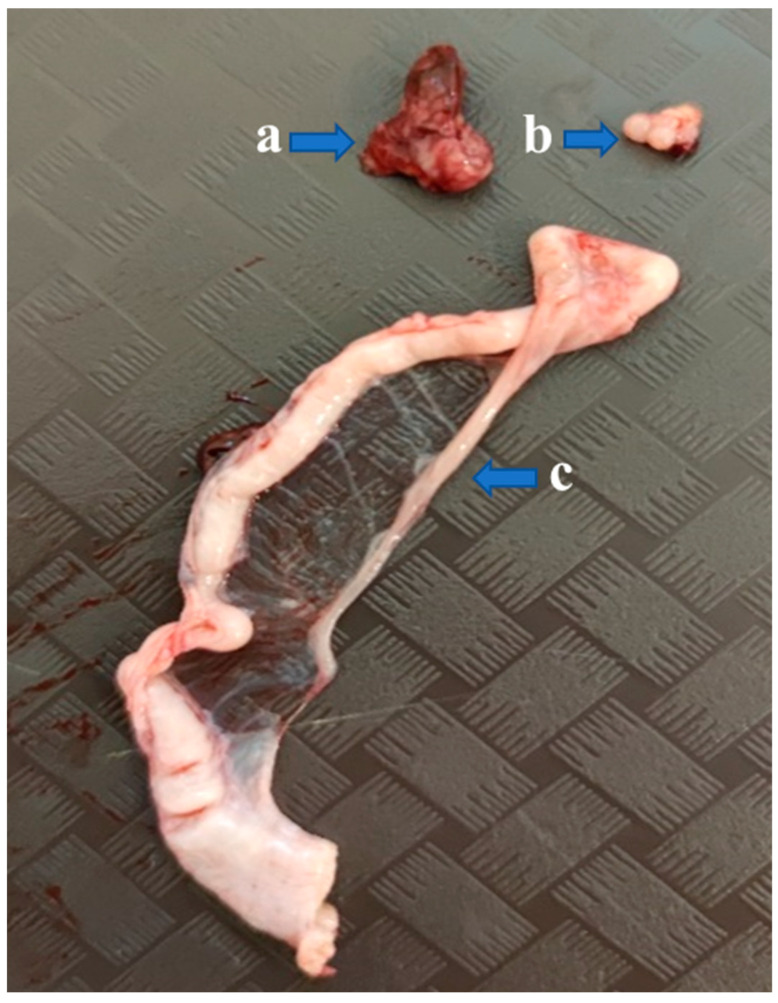
Gonads of a recipient Mulard duck with an adhered donor ovary. The recipient’s ovary is atrophied (a), while developing follicles can be seen on the donor ovary (b), and the oviduct is developed (c) as well.

Necropsies of the operated recipients were performed at 52 weeks of age; the adhesion rate of the grafted donor ovaries was 15% in the Pekin donor/Mulard recipient group without GnRH treatment. In the buserelin-treated group, the adhesion rate of the donor ovaries was 31%. In the operated, non-treated group, there were no developing follicles or oviducts. However, in the buserelin-treated, operated group, in 25% of the recipients with adhered donor ovaries, primordial follicles and developed oviducts were found (Figure 9).

#### 3.3.2. Results of the Hormonal Level Determination

**Figure 10:**
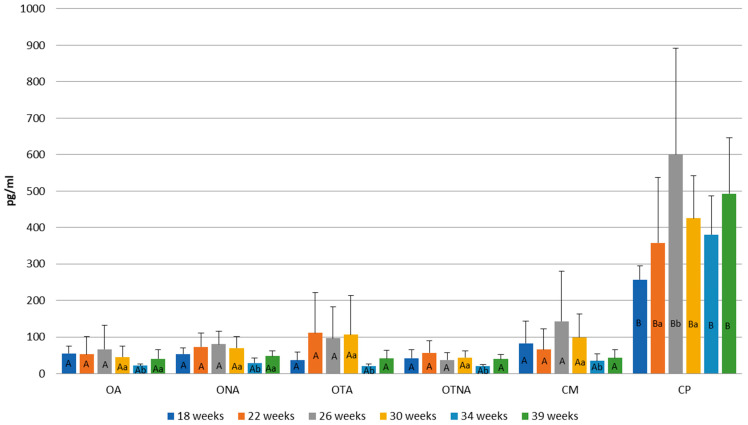
Comparison of estrogen levels at 18, 22, 26, 30, 34, and 39 weeks of age in operated Mulard recipients with adhered donor ovary (OA) and non-adhered donor ovary (ONA); operated recipients treated with GnRH analogue with adhered donor ovary (OTA) and non-adhered ovary (OTNA); and control Mulard (CM) and control Pekin (CP) groups. A, B: *p* ≤ 0.05: significance between the groups. a, b: *p* ≤ 0.05: significance within the groups.

Changes in the estrogen levels between the groups at six different sampling times are presented in Figure 10. In the control Pekin group (CP), estrogen levels were consistently higher at all examined time points compared to the other groups (CP: 256.67 pg/mL–600 pg/mL; other groups: 20.19 pg/mL–142.97 pg/mL). Within-group analysis revealed mild fluctuations in estrogen levels in the operated adhered group (OA), with the lowest concentration observed at 34 weeks of age (22.06 pg/mL). A similar trend was noted in the operated non-adhered group (ONA). In the treated adhered group (OTA), peak estrogen levels occurred at 22, 26, and 30 weeks (111.79 pg/mL, 96.81 pg/mL, and 106.15 pg/mL, respectively), followed by a marked decline at 34 weeks (20.9 pg/mL). The treated non-adhered group (OTNA) also exhibited the lowest estrogen concentration at 34 weeks (20.19 pg/mL). In the control Mulard group (CM), estrogen levels fluctuated, with the lowest values recorded at 34 and 39 weeks (34.93 pg/mL and 43.35 pg/mL, respectively). In contrast, the estrogen levels in the control Pekin group (CP) were the lowest at 18 weeks and the highest at 26 weeks of age (256.67 pg/mL and 600.63 pg/mL, respectively).

**Figure 11:**
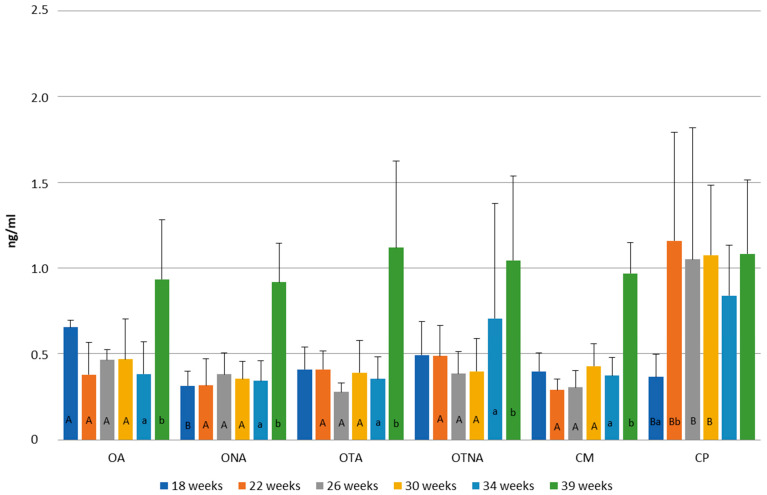
Comparison of progesterone levels at 18, 22, 26, 30, 34, and 39 weeks of age in operated Mulard recipients with adhered donor ovary (OA) and non-adhered donor ovary (ONA); operated recipients treated with GnRH analogue with donor ovary (OTA) and non-adhered ovary (OTNA); and control Mulard (CM) and control Pekin (CP) groups. A, B: *p* ≤ 0.05: significance between the groups. a, b: *p* ≤ 0.05: significance within the groups.

Figure 11 shows the progesterone level changes in the groups at 18, 22, 26, 30, 34, and 39 weeks of age. At week 18, the operated adhered group (OA) exhibited higher progesterone levels compared to both the operated non-adhered (ONA) and control Pekin (CP) groups (OA: 0.66 ng/mL, ONA: 0.31 ng/mL, CP: 0.37 ng/mL). At weeks 22, 26, and 30, the control Pekin group (CP) consistently showed the highest progesterone levels among all groups (CP: 1.05 ng/mL–1.16 ng/mL; other groups: 0.28 ng/mL–0.49 ng/mL). With the exception of the control Pekin group (CP), all other groups reached their peak progesterone levels at week 39 (OA: 0.93 ng/mL, ONA: 0.92 ng/mL, OTA: 1.12 ng/mL, OTNA: 1.05 ng/mL, CM: 0.97 ng/mL). In the control Pekin group (CP), the lowest progesterone concentration was measured at week 18, while levels fluctuated at subsequent time points correlating with the pattern of egg production.

## 4. Discussion

In domestic ducks, interspecific ovarian tissue transplantation (in a Muscovy duck donor/Pekin duck recipient combination) was reported by Song and Silversides [12]; 25% of the donor gonads adhered, and both donor-derived Muscovy duck offspring and recipient-derived Mulard duck offspring were produced. In our study, in which a well-established surgical technique was applied to Mulard ducks as sterile recipients, the adhesion rates of the grafted native and frozen/thawed ovaries were similar (ranging from 15% to 66%). However, while the ovulation process began in 16% of the recipients in the second experiment, the eggs did not move into the oviduct, but into a separate serous membrane capsule. In spite of the infertility and decreased hormonal production of Mulard ducks, both developed follicles on the adhered donor ovaries and developed oviducts were found. Based on these results, we question whether folliculogenesis and oviduct development can be influenced by hormonal treatment. Hanafy et al. [10] applied buserelin acetate, a synthetic GnRH analogue, to TETRA SL non-laying hens. Synthetic GnRH analogues have a high receptor-binding affinity, which results in a longer-lasting effect compared to non-synthetic forms. Because of their stronger stimulation of gonadotropin release and their physiological effects, they are suitable for treating reproductive dysfunctions [13]. The buserelin treatment used in our study was based upon the doses applied by Hanafy et al. [10], but due to the larger body weight of Mulard ducks, we increased the dose to 200 microliters. As egg production begins at 22 weeks of age in the control Pekin layer group, we began the GnRh analogue treatment at this stage. Interestingly, lower estrogen levels were observed in the operated groups in Experiment 3 compared to Experiment 2. This difference may be attributable to the more pronounced follicular development seen in Experiment 2. Supporting this explanation, similar estrogen levels were recorded in both the adhered and non-adhered operated groups. As the Mulard is an interspecific hybrid that needs to be recreated each season, genetic variability among individuals is likely. This diversity could impact the efficiency of transplanted ovarian tissue adhesion, hormone production, and subsequent follicular development and ovulation. In our study, we focused on measuring estrogen and progesterone levels, which are primarily secreted by developing follicles. Consequently, further investigations into potential differences in other components of the hypothalamo–hypophyseal–gonadal axis are needed. Notably, in Experiment 3, both the extent and quality of follicular development in successfully adhered ovarian tissue grafts were diminished compared to Experiment 2. Such findings may suggest hormonal imbalance, as the process of vitellogenesis is closely dependent on the coordinated function of the hypothalamo–hypophyseal axis [14,15]. In animals treated with a GnRH analogue at 22 weeks of age where the grafted ovaries successfully adhered, estrogen levels increased at 22, 26, and 30 weeks, suggesting a possible effect of the GnRH analogue treatment in spite of the absence of successful ovulation. This raises the question of whether the timing and dosage of the GnRH analogue treatment were appropriate—an issue that warrants further investigation. Administering higher doses of buserelin may have an adverse effect on reproductive traits [16]. Based on the current findings, GnRH analogue treatment did not influence progesterone levels. Future studies could also measure the LH and FSH levels and determine the number of estrogen receptors in the organs of Mulard ducks, as estrogen 2 receptors were found to be associated with egg weight in the hypothalamus, hypophysis, ovary, and different sections of the oviduct in Leizhou ducks [17].

## 5. Conclusions

In the case of ovarian tissue transplantation, the use of sterile recipients may be a promising option from a gene preservation perspective, but it still requires further research to become practically applicable. Understanding the neuroendocrine regulation of ovulation in other bird species could be useful for addressing specific issues in reproductive biology. Our results raise several important questions. For instance, how can ovulation into the oviduct be ensured in these animals, and is this issue fundamentally hormonal in origin? How might the functional capacity of donor gonads be enhanced? Finally, is the Mulard, as a sterile hybrid, truly suitable for egg production?

## Data Availability

The raw data supporting the conclusions of this article will be made available by the authors on request.
